# Simulation of the Continuous Casting and Cooling Behavior of Metallic Glasses

**DOI:** 10.3390/ma10040420

**Published:** 2017-04-17

**Authors:** Zhipu Pei, Dongying Ju

**Affiliations:** 1 Graduate School of Saitama Institute of Technology, Fusaiji 1690, Fukaya 369-0293, Japan; peizhipu@gmail.com; 2 High-Tech Research Center, Saitama Institute of Technology, Fusaiji 1690, Fukaya 369-0293, Japan; 3 University of Science and Technology Liaoning, Anshan 114051, China

**Keywords:** metallic glass, critical cooling rate, twin-roll casting, simulation

## Abstract

The development of melt spinning technique for preparation of metallic glasses was summarized. The limitations as well as restrictions of the melt spinning embodiments were also analyzed. As an improvement and variation of the melt spinning method, the vertical-type twin-roll casting (VTRC) process was discussed. As the thermal history experienced by the casting metals to a great extent determines the qualities of final products, cooling rate in the quenching process is believed to have a significant effect on glass formation. In order to estimate the ability to produce metallic glasses by VTRC method, temperature and flow phenomena of the melt in molten pool were computed, and cooling rates under different casting conditions were calculated with the simulation results. Considering the fluid character during casting process, the material derivative method based on continuum theory was adopted in the cooling rate calculation. Results show that the VTRC process has a good ability in continuous casting metallic glassy ribbons.

## 1. Introduction

Amorphous metallic alloys exhibit outstanding mechanical, magnetic, and chemical properties that are unusual for solid metals. More than half a century has passed since Pol Duwez reported his remarkable findings in 1960 [1], and the subject area has moved a long way. Once the potential of metallic glasses for any certain application was recognized, it was seen that a scaling-up method was needed to deploy metallic glasses commercially [2]. To produce metallic glasses continuously, in the early 1970s, researchers at Allied Chemical Corporation developed continuous casting or melt spinning processes for commercial manufacture of metallic glass ribbons and sheets, and at that time the field of melt spinning technique in producing metallic glasses started gaining momentum [3,4,5,6,7]. Relations between processing parameters and ribbon quality and dimensions have been discussed by some researchers in recent years [8,9,10,11]; however, only a small fraction of the metallic glasses have so far been commercially deployed mainly due to the difficulty in obtaining good quality in such thin products. For applications in transformer cores, metallic glasses such as Metglas^®^ Alloy 2605SA1 can reduce transformation losses by 70% compared to crystalline iron cores [2,12].

In the melt spinning process, conditions between the roll-contact surface and free surface of ribbon are different, and these will affect the final ribbon quality [9,13]. In contrast, metallic ribbons produced by twin-roll casting technique are casted and hot rolled at the same time and a better surface quality is expected. The concept of continuous casting in a single stage dates back to Bessemer, in 1846, who envisioned using a twin-roll technique to cast steel strips [14], but technical realization was more than a century later. In 1970, after Duwez’s seminal discoveries, a twin-roll casting (TRC) technique for preparing uniform films of metastable phases was devised by Chen and Miller [15]. To date, this technique in producing metallic glass ribbons is still almost limited to laboratory scale studies [16,17,18,19,20,21,22]. It turns out that TRC is an available process for producing amorphous alloy sheets with a wide range of cooling rates. Nevertheless, most of the studies so far have been based on horizontal type twin-roll casters. Studies on TRC technique for producing metallic glasses are listed in Table 1.

Because the influence of gravity, it shows that the casting speed of vertical type twin-roll casting (VTRC) is higher than horizontal-type twin-roll casting (HTRC), and the heat transmission of the former is more effective [24,25,26]. In the present study, based on the VTRC process, thermal history experienced by metallic materials during casting process was computed, and the cooling rate was also calculated. In order to make a judgment on the process cooling ability in producing metallic glasses, a continuous cooling transformation (CCT) diagram was proposed. The influences of casting parameters on glass forming were discussed. As Al-rich metallic glasses are interesting lightweight alloys with a high specific strength (compressive fracture strength exceeded 1000 MPa) which make them a potential material for aerospace applications [27,28], material property parameters of high aluminum alloys (Al contents as high as 35–40 at %, e.g., Al_35_La_50_Ni_15_) are adopted in this paper.

## 2. TRC Simulation

**Figure 1:**
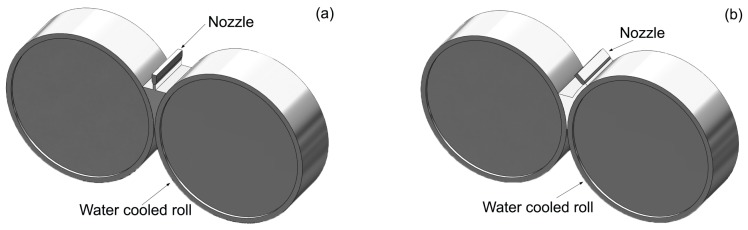
Schematic of TRC process: (**a**) Center pouring mode; (**b**) One side pouring mode.

Amorphization or crystallization can occur depending on the cooling rates and thermal behavior experienced by the melt [17,25]. For a better understanding of the solidification behavior of TRC process, thermal flow simulation was carried out based on the condition of a pilot vertical twin-roll caster with roll radius of 150 mm, roll width of 100 mm. Figure 1 shows the schematic of TRC process, with melt supplied in two ways (i.e., center pouring mode shown in Figure 1a, and one side pouring mode shown in Figure 1b). Because the ratio of the width to thickness is quite large, if one ignores the effect of side dams, a 2D finite element method (FEM) model is used. Considering the high speed feeding flow of liquid metal during the strip casting process, flow phenomenon in the molten pool is characterized as turbulent flow. Moreover, the following assumptions are made for steady-state simulations: there is no relative slip between the roll and strip, and heat transfer coefficient between them is constant (i.e., 18 kW∙m^−2^∙K^−1^); surface temperature of the roller is 373 K; free surface of the melt is steady. Influences of melt level *h*, strip thickness *δ*, casting speed *v* (i.e., rotating speed) and pouring temperature *T_p_* are considered in simulation. Considering geometric symmetry of the center pouring mode (i.e., Figure 1a), half of the twin-roll casting model was employed.

**Figure 2:**
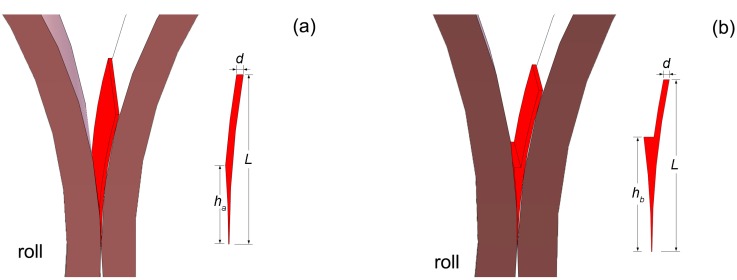
Schematic of the molten pool of one side pouring mode: (**a**) *h_a_* depends on the roll gap and *d*; (**b**) *h_b_* > *h_a_* and can change independently.

Figure 2 illustrates the formation of the molten pool of the one side pouring mode. The process and dimensions are specified as follows: the melt is ejected on one roll through a nozzle, a strip shell forms along the roll surface, and the strip shell thickness is marked as *d*, which has the same value as nozzle. The metal shell of this part is cooled by the roll (the metal-roll contact side) and the atmosphere around it (the metal-air contact side), nearly the same as melt spinning process. As the metal shell rolling down towards to the nip, it contacts to the other side roll, and the height to the nip from this location is marked as *h*. The metal of this part is cooled by both of the rolls. In Figure 2a, *h*, which is marked as *h_a_*, depends on the metal shell thickness *d* and the roll gap. We can get a larger value of *h* with a smaller roll gap under a certain metal shell thickness *d*. In Figure 2b, a molten liquid level has formed, and *h* here is marked as *h_b_*, which is higher than *h_a_* under the same metal shell thickness and roll gap, and can change independently. With the effect of roll pressure (i.e., roll separating force), a thin solidified strip forms and escapes from the roll nip. The total height of the molten pool is marked as *L*.

## 3. Calculation of Time Reduced Temperature Transformation (T-T_r_-T) and CCT Diagrams

### 3.1. Calculation of T-T_r_-T Diagram

The following equation based on the steady-state homogeneous nucleation theory proposed by Davies and derived by Inoue [29]:

(1) $$t=\left[\frac{31}{k}{\left(\frac{xa_{0}^{9}}{N_{v}}\right)}^{1/4}\right]\times \left[\frac{1}{T_{r}\Delta T_{r}^{3/4}}\exp\left(\frac{0.268}{T_{r}^{3}\Delta T_{r}^{2}}\right)\exp\left\{\frac{2}{5}{\left(\frac{T_{r}}{\Delta T_{r}}\right)}^{3/4}\right\}\right]\times \left[\eta_{0r}\exp\left(\frac{B_{r}}{T_{r}-T_{0r}}\right)\right]$$

Equation (1) gives a time transformation curve (C-curve) that expresses the time to transform to crystal as a function of temperature. Here, *k* is the Boltzmann constant, *x* is the fraction of crystal formed in time *t*, and a volume fraction of 10^−6^ as a just-detectable concentration of crystals was used [30]. *a_0_* is the mean atomic diameter, *N_v_* is the volume concentration of atoms. *T_r_* (=*T/T_m_*) is the reduced temperature, and Δ*T_r_* (=1 − *T_r_*) is the reduced undercooling of the melt. *B_r_*, *T*_0*r*_ and *η*_0*r*_ are defined as *B/T_m_*, *T*_0_*/T_m_* and *η*_0_*/T_m_*respectively. *B*, *T*_0_ and *η*_0_ are the parameters in the Vogel-Fulcher-Tammann (VFT) equation:

(2) $$\eta \left(T\right)=\eta_{0}\exp\left(\frac{B}{T-T_{0}}\right)=\eta_{0}\exp\left(\frac{D^{*}T_{0}}{T-T_{0}}\right)$$

where *T*_0_ is known as an ideal glass transition temperature and *B* is constant depending on materials. D* is the fragility parameter (1 ≤ D* ≤ 100). *η*_0_ has the relation of *η*_0_* = N_A_h/V*, with *N_A_*, Avogadro’s constant, *h*, Planck’s constant and *V*, the molar volume [31]. In this paper, the values of *V* are calculated by the JMatPro software and then we can get the value of *η*_0_. The first term of Equation (1) is considered a constant [29], in this paper it is 0.89 × 10^−6^ J^−1^∙K∙m^3^. Since *B* and *T*_0_ are empirical parameters and were not known clearly for the alloys below, referring to the data summarized by Takeuchi and B.A. Sun [27,29,32], considering the mostly used processing method of high aluminum content bulk metallic glasses [2,33,34], parameters of hypothetical alloys used in this paper are listed in Table 2. These hypothetical alloys are assumed to have compositions range near the given alloy (i.e., Al_35_La_50_Ni_15_).

### 3.2. Calculation of CCT Diagram

In order to determine the critical cooling rate for a glass-forming material solidified from liquid state without crystallization, constructing a CCT curve instead of time-temperature-transformation (TTT) curve is more realistic [35]. Here, we used the additivity rule to relate the transformation behavior during continuous cooling with the isothermal transformation data calculated above. The additivity rule was proposed by Scheil firstly and can be expressed by [36]:

(3) $$\sum_{i=1}^{n}\frac{t_{i}}{\tau_{i}}=1$$

where *t_i_* is the time spent at a particular temperature and *τ_i_* is the incubation time at that temperature. According to the additivity rule, CCT curve could be gained using the calculated T-T_r_-T diagram. The cooling routes used in calculation are given by:

(4) $$T\left(t\right)=T_{m}+qt$$

where *q* is the cooling rate, and here it is negative.

## 4. Results and Discussion

### 4.1. Estimation of the Critical Cooling Rates Using CCT Curves

**Figure 3:**
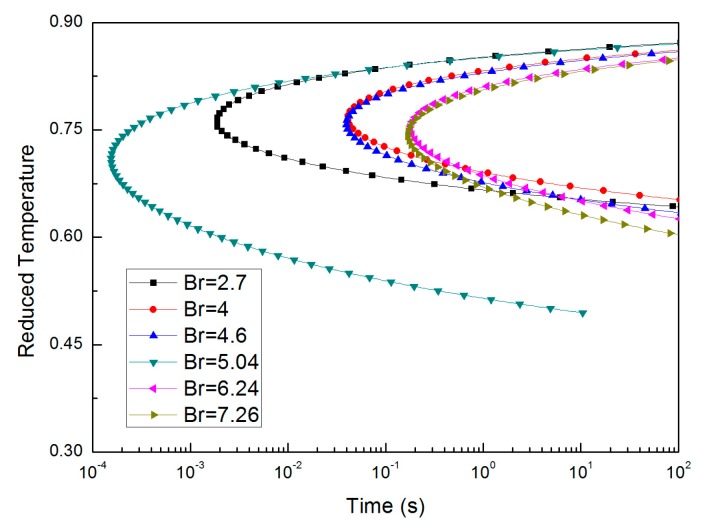
Calculated T-Tr-T diagram: alloys with compositions close to Al_35_La_50_Ni_15_.

It was found that strong glass formers exhibit a very small *T*_0_ and a very high melt viscosity, while fragile glass formers, show a *T*_0_ near *T_g_*, as well as low melt viscosities [37]. The T-T_r_-T curves of Al-rich alloys listed in Table 1 calculated by Equation (1) are shown in Figure 3. Alloys with compositions close to Al_35_La_50_Ni_15_ could have small D* and *T*_0_ near *T_g_*, and they may have “C-curves” located between the area of the two curves which with *B_r_* of 2.7 and 4. The curve with *B_r_*of 2.7 has a “nose time” of 1.9 × 10^−3^ s at *T_r_* of 0.76 and a critical cooling rate of 1.22 × 10^5^ K/s. The curve with *B_r_* of 4 has a “nose time” of 0.043 s at *T_r_* of 0.75 and a critical cooling rate of 5.55 × 10^3^ K/s. Therefore, according to the T-T_r_-T curve, a critical cooling rate of four orders of magnitude was estimated for these alloys.

**Figure 4:**
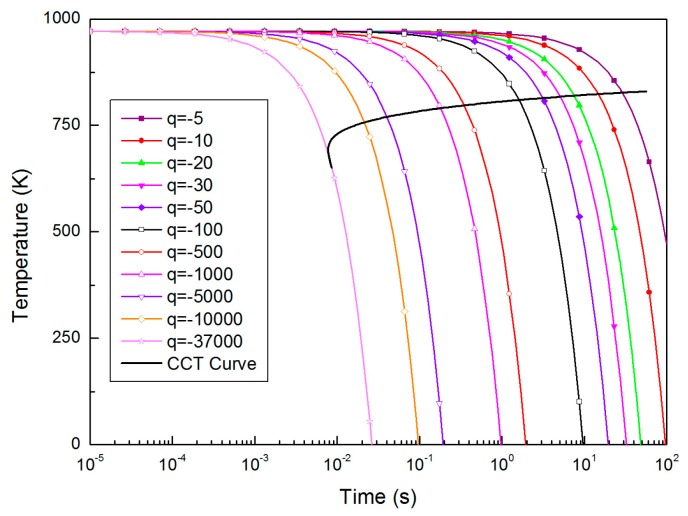
Calculated continuous cooling transformation (CCT) diagram: alloy with composition close to Al_35_La_50_Ni_15_.

Figure 4 shows the calculated CCT curve of the alloy which has composition close to Al_35_La_50_Ni_15_ with *B_r_* of 2.7. It can be found that the critical cooling rate of the hypothetical alloy is 3.71 × 10^4^ K/s, and it is lower than the value attained from T-T_r_-T diagram.

### 4.2. Influence of Casting Parameters

**Figure 5:**
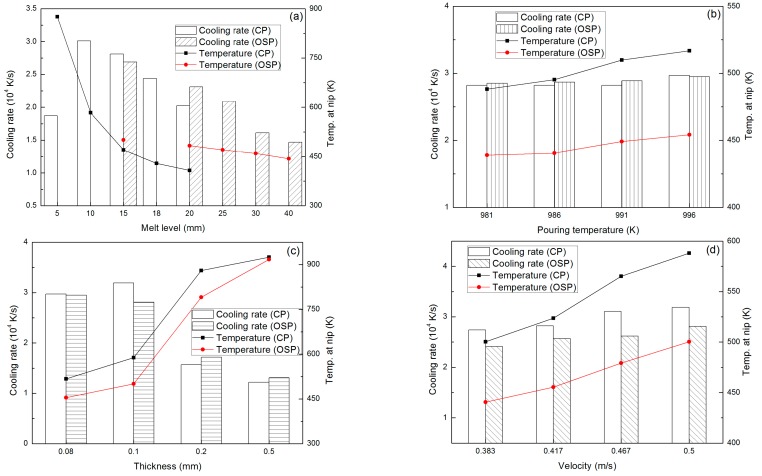
Effects of casting conditions on cooling rate: (**a**) Effect of melt level; (**b**) Effect of pouring temperature; (**c**) Effect of strip thickness; (**d**) Effect of casting speed.

In the evaluation of the cooling ability of the twin-roll caster, average cooling rate, *R_AVG_*, was calculated with the following equation:

(5) $$R_{AVG}=\frac{\Delta T}{\Delta t}\approx \frac{T_{p}-T_{nip}}{h}\times v_{c}$$

where *T_p_* and *T_nip_* are, respectively, pouring temperature and temperature at the roll nip, *h* the melt level of molten pool, and *v_c_* the casting speed. Figure 5 shows the influences of different casting conditions on the average cooling rate for Al_35_La_50_Ni_15_ alloy based on simulated results, and CP indicates the center pouring mode, OSP indicates the one side pouring mode. For the OSP mode, molten pool has a shape shown in Figure 2a. Here, we call *L* the melt level. From these results we can see that the average cooling rate under the CP mode is faster than that under the OSP mode. Figure 5a shows the results under the casting conditions of pouring temperature 918 K, casting speed 0.5 m/s and strip thickness 0.08 mm. We can see that average cooling rate becomes slower with the increasing of melt level. Well, for the melt level of 5 mm, it is too fast to solidify within such a short metal-roll contact distance that temperature at the roll nip is 876.11 K, and the process will not continue. When the strip thickness (i.e., nip width) comes to 0.2 mm and 0.5 mm with a melt level of 10 mm, as shown in Figure 5c, much more heat needs to be dissipated for the liquid to become fully solidified before it passes the roll nip, and the process cannot be continued. On the other hand, when setting the melt level smaller and strip thickness thinner, a faster cooling rate could be attained and temperature at the roll nip is also appropriate. However, it may cause a rolling block if the temperature at the roll nip gets too low since a fully amorphous metal is formed before it passes the roll nip. From Figure 5b we can see that there is no influence of the pouring temperature on the average cooling rate, but it can adjust the temperature at roll nip, a lower pouring temperature leads to a lower nip temperature. As a faster casting speed causes a faster cooling rate and a higher nip temperature (Figure 5d), we can control the nip temperature through adjusting the pouring temperature. However, if the nip temperature is so high that it makes the temperature of the supercooled alloy still well above *T_g_* before it passes the roll nip area, full formation of an amorphous structure might not be possible [17], therefore the casting speed has an upper limit. Incidentally, the cooling rates at current conditions, as illustrated in Figure 5b,d, are all in the order of magnitude of 10^4^ K/m, which has a same level with the calculated critical cooling rate.

Based on the continuum theory, a special form of material (or material-time) derivative for any physical quantity *ψ* under motion status in Eulerian coordinate has been proposed by Ju [38]:

(6) $$\dot{\psi }=\frac{D\psi }{Dt}=\frac{\partial \psi }{\partial t}+v\times \frac{\partial \psi }{\partial x}$$

where *ν* is the velocity vector of material point, ***x*** is a vector and represents a space-fixed coordinate system. According to the theory above, considering the thermal phenomenon of the molten pool, cooling rate *R*(*T*) at the center of pool was calculated by:

(7) $$R\left(T\right)=\frac{DT}{Dt}=\frac{\partial T}{\partial t}+\nu \times \frac{\partial T}{\partial y}$$

where *T* is the temperature at pool center, *y* is the location in the pool height direction, *ν* is the velocity of the local melt, and *t* is the time.

According to the analyses of results in Figure 5, in the twin-roll casting process, one of the most important factors is the melt level *L*. The influences to the temperature distribution under different conditions were discussed as below. Casting conditions are as follows: pouring temperature 981 K, casting speed 32 m/min, strip thickness 0.1 mm, and melt levels under the two pouring mode are listed in Table 3. For the one side pouring mode (Figure 2b), it has a strip shell thickness of 1 mm (i.e., d = 1 mm).

It can be seen from Table 3 that, for a certain melt level, we can get a faster cooling rate with using the CP mode, and also nip temperature is lower than that under OSP mode. On the other hand, however, it is not expected to get a temperature too low at the roll nip because the special properties of metallic glass. Fully solidified metallic glass deforms hardly due to its very high strength. Therefore, we can use the OSP mode to adjust the strip temperature at nip for a certain melt level.

## 5. Conclusions

1. A CCT diagram of a certain alloy could be attained by the combination of an equation derived by Inoue and the additivity rule.
2. Critical cooling rate of a metallic alloy for forming metallic glass could be evaluated using the current method.
3. Changing the casting conditions or adopting the one side pouring mode could improve the temperature distribution of the pool metal, and the rolling block can be avoided in the VTRC process.
4. Cooling rates with 4 orders of magnitude by the VTRC process under the current conditions can be attained, which shows that the VTRC technique has a potential ability in continuous fabrication of Al-rich bulk metallic alloys in sheet form.

## Figures and Tables

**Table 1:** Papers on the research of twin-roll casting (TRC) of metallic glasses.

Year	Type	Cooling Rate (K/s)	Speed (rpm)	References
1970	Vertical	10^5^	100–5000	[15]
1974	Vertical	10^5^	---	[16]
2005	Horizontal	10^2^–10^3^	1, 3	[17,18,23]
2007	Horizontal	---	0.75 m/s	[20]
2010	Horizontal	---	102 m/s	[22]
2013	Vertical	1–10^3^	---	[21]

**Table 2:** Parameters used for the calculation of T-Tr-T diagram for aluminum alloys.

Alloy	*η*_0_ (Pa·s)	*B* (K)	*B_r_*	D*	*T*_0_ (K)	*T*_0*r*_	*T_m_* (K)	*x*
Al_35_La_50_Ni_15_/Hypothetical alloys	2.39 × 10^−5^	4893.84	5.04	18	271.88	0.28	971	10^−6^
		7049.46	7.26	22	320.43	0.33		
		6059.04	6.24	16	378.69	0.39		
		4466.6	4.6	10	446.66	0.46		
		3884	4	8	485.5	0.5		
		2621.7	2.7	5	524.34	0.54		

**Table 3:** Influence of melt level to cooling rates and nip temperature under the two pouring modes.

Melt Level/L (mm)		11.63	15	20	25	30	35
Cooling Rate (10^4^ K/s) R (737.96)	CP	3.24	3.88	3.27	38	39.6	---
	OSP	---	3.53	2.77	3.59	2.21	2.74
*T_nip_* (K)	CP	497.16	445.27	405.11	396.25	395.44	---
	OSP	---	470.54	501.07	458.26	423.21	417.58
